# The Potential Use of Vitamin C to Prevent Kidney Injury in Patients with COVID-19

**DOI:** 10.3390/diseases9030046

**Published:** 2021-06-28

**Authors:** Feng Xu, Yawei Wen, Xinge Hu, Tiannan Wang, Guoxun Chen

**Affiliations:** 1 Department of Urology, The First Affiliated Hospital of Zhengzhou University, Zhengzhou 450052, China; xufeng6602@163.com (F.X.); wenyawei1516@163.com (Y.W.); 2 Department of Nutrition, University of Tennessee at Knoxville, Knoxville, TN 37996, USA; xhu25@vols.utk.edu (X.H.); twang14@vols.utk.edu (T.W.)

**Keywords:** COVID-19, vitamin C, acute kidney injury, oxidative stress, inflammatory cytokine storm

## Abstract

The newly found SARS-CoV-2 has led to the pandemic of COVID-19, which has caused respiratory distress syndrome and even death worldwide. This has become a global public health crisis. Unfortunately, elders and subjects with comorbidities have high mortality rates. One main feature of COVID-19 is the cytokine storm, which can cause damage in cells and tissues including the kidneys. Here, we reviewed the current literature on renal impairments in patients with COVID-19 and analyzed the possible etiology and mechanisms. In addition, we investigated the potential use of vitamin C for the prevention of renal injury in those patients. It appears that vitamin C could be helpful to improve the outcomes of patients with COVID-19. Lastly, we discussed the possible protective effects of vitamin C on renal functions in COVID-19 patients with existing kidney conditions.

## 1. Introduction

Coronaviruses have received their name due to their appearance under an electron microscopy. They infect both humans and animals. Their infections in humans lead to clinical symptoms in the respiratory, digestive, and central nervous systems. The impact on the respiratory system is the most obvious, which can result in death [1]. At the end of 2019, a pandemic of a respiratory disease of unknown origin occurred in Wuhan, China. Some patients died of respiratory distresses. The pathogen was finally determined as a novel type of coronavirus after its sequence was revealed in January 2020. The World Health Organization (WHO) named this coronavirus as the severe acute respiratory syndrome coronavirus 2 (SARS-CoV-2) belonging to the β-coronavirus cluster, which also contains members responsible for severe acute respiratory syndrome (SARS) and Middle East respiratory syndrome (MERS) pandemics [2]. Then, the disease associated with this SARS-CoV-2 infection was named as coronavirus disease 2019 (COVID-19). According to the earliest available data, the majority of patients diagnosed with COVID-19 before 1 January 2020, were linked to a seafood wholesale market in Wuhan [3]. Around the world, as of 15 April 2021, according to WHO (https://www.who.int/emergencies/diseases/novel-coronavirus-2019), more than 137,866,311 COVID-19 cases have been reported, and the death toll has exceeded 2,965,707 in 223 countries, areas or territories.

In the initial phase of the SARS-CoV-2 infection, the latent period is about 4.5 days, and the infectivity is high, with the number of infected patients doubling at 7.4 days [3]. Viral particles in the air enter subjects via the mucous membranes in the respiratory tract, oral cavity, or eye conjunctiva, etc. The most common symptoms are fever and dry cough, which can be seen in 80 to 90% of patients with COVID-19 [4]. Additionally, 40% of COVID-19 patients suffer from fatigue and 18.6% experience dyspnea, while nasal congestion, nausea, and diarrhea are seldom reported [5]. Clinical data indicate that one out of six patients with COVID-19 will develop respiratory distresses and will need adequate medical care [6]. Before people receive vaccines, the best way to prevent the spread of this virus is to maintain social distances, wash hands frequently, avoid close contact, and do a good job of early screening and isolation of infected people.

Vitamin C (ascorbic acid) is a micronutrient that is water soluble and considered to be an antioxidant [7]. It influences various aspects of the immune system, particularly immune cell functions [8,9]. The human body cannot synthesize vitamin C due to the lack of the enzyme responsible for the last step of its de novo biosynthesis [10,11]. Vitamin C deficiency leads to scurvy, a disease characterized by the inability to form functional collagen, which causes weakening of collagen structures in tissues, poor wound healing, and impaired immunity [12]. Individuals with scurvy are highly susceptible to potentially fatal infections such as pneumonia [13]. In addition, infections can significantly increase vitamin C usage due to inflammation and metabolic requirements. It has been observed that scurvy has often followed infectious epidemics in populations and may develop after respiratory infections [14]. This may be apparent in malnourished individuals.

## 2. The Impacts of the Cytokine Storm in COVID-19

Cytokines are signaling proteins produced by immune cells to regulate the body’s immune responses [15,16,17]. Those immune cells include neutrophils, monocytes, macrophages, as well as B and T cells, which synthesize and secrete cytokines to modulate immune responses in animals [18,19].

Cytokine release syndrome (CRS), also known as ‘cytokine storm’, can occur in various conditions including sepsis, severe systemic infection, and chimeric antigen receptor T cell therapy [20]. The extensive and uncontrolled release of proinflammatory cytokines is detrimental to the body. Clinically, cytokine storm is often associated with systemic inflammation and multiple organ failures [11]. Patients with COVID-19 in critical conditions have elevated cytokine profiles similar to those in patients with SARS and MERS [21]. The levels of interleukin (IL)-1α, IL-1β, IL-7, IL-8, IL-9, IL-10, granulocyte-macrophage colony stimulating factor, interferon gamma (IFN-γ), fibroblast growth factor, granulocyte-colony stimulating factor (G-CSF), IFN-γ-inducible protein (IP10), macrophage inflammatory protein 1 alpha (MIP1A), platelet-derived growth factor, monocyte chemoattractant protein (MCP1), vascular endothelial growth factor, and TNF-α of inflammatory factors are increased in patients with COVID-19 [22]. In addition, elevated levels of IL-6 have been observed in patients with COVID-19, which may be considered to be a biomarker for predicting disease severity [23,24]. A large retrospective cohort study has also reported an association between IL-6 and mortality in patients with COVID-19 [25].

## 3. The Effects of COVID-19 on the Kidneys and Treatment Strategies

The occurrence of CRS has been observed in COVID-19 since it was reported [26]. In patients with COVID-19, the coronavirus infection triggers an inflammatory cytokine storm. The significant elevations of cytokines in the circulation cause serious inflammatory reactions, and also different degrees of organ damage, resulting in the corresponding symptoms [27]. After reaching the kidneys, inflammatory cytokines cause renal tubular damage, affect the filtration of the kidneys, lead to the accumulation of metabolites in the body, and further aggravate the clinical symptoms and threaten life [28].

Cytokines mainly damage the renal tubules and cells. The damage alters renal tubular permeability, which leads to impaired renal filtration function and renal injuries. When CRS occurs, a large number of cytokines circulate in the blood and damage the vascular permeability. For example, the levels of IL-2, IL-6, IL-7, IL-10, IP10, G-CSF, MCP1, MIP1A, and TNF-α are higher in critically ill patients with COVID-19 than those in the mild group [27,29]. Intravascular fluids infiltrate into the interstitial space, which cause a relative lack of blood volume and systemic edema. The reduced blood volume result in a drop in blood pressure and cause insufficient renal blood supply. The filtration rate of the kidneys decreases, which further causes accumulation of harmful substances in tissues, intensifies systemic symptoms, and causes a vicious cycle to aggravate the conditions. If renal damage is not corrected, further development will lead to acute renal failure in patients, especially the elderly or those with basic diseases.

The prevalence of acute kidney injury (AKI) among patients with COVID-19 was initially thought to be low. For example, in a Chinese cohort of 1099 patients with COVID-19, 93.6% were hospitalized, 91.1% had pneumonia, 5.3% were admitted to the ICU, 3.4% had acute respiratory distress syndrome and only 0.5% had AKI [4]. However, a recent study indicated that the incidence of AKI in 85 cases of COVID-19 patients was reported to be about 27.6% [30]. It is easier to develop acute renal failure in elderly patients. Severe acute tubular necrosis and lymphocytic infection were found in the autopsies of six subjects who died with AKI, but cortical necrosis was not known [30]. The human kidneys may be a target for coronavirus infection [31]. The autopsy report of a patient who died of COVID-19 also revealed acute proximal tubule injury herniation, renal tubular endodermal injuries, peripheral erythrocyte aggregation, glomerular fibrin embolism, and inflammation [32]. Some of these patients did not show evidence of AKI detected by routine tests (creatinine and/or urea nitrogen), which indirectly suggested that early renal injuries may be overlooked clinically.

So far, the incidence of renal injuries in patients with coronavirus infection has not been reported clinically. This may be due to the fact that the disease progresses rapidly after a patient is infected with the virus, and impacts the lungs, brain and heart first. Early effects on the kidneys do not appear. Probably, only microscopic changes occur in the early stage of kidney injury [33]. However, after the infection of SARS-CoV-2, the disease progresses rapidly in the respiratory system, and it becomes difficult to detect damage to the renal functions over time [33]. Therefore, the changes in the kidneys tend to be ignored due to the dramatic changes in other organs. We argue that it is probably too late to correct and protect the kidney functions when the damage has occurred. As the kidneys play a critical role in the regulation of whole-body metabolism and homeostasis, the damage to renal functions will affect the whole-body metabolism and aggravate the patient’s condition. Early detection and protection of renal functions in patients with COVID-19 should be planned ahead of time and considered to be serious during the treatment of COVID-19.

For COVID-19 patients with kidney conditions, additional treatments probably have to be used. Extracorporeal “blood purification,” mainly in the form of hemodialysis, has been a main clinical practice of many nephrologists for the past five decades [28]. Another possibly older procedure, therapeutic plasma exchange, separates, and then removes potential detrimental materials from the plasma of the patients [34,35,36]. In contrast to hemodialysis, therapeutic plasma exchange preferentially removes biologic substances of high molecular weight such as autoantibodies or alloantibodies, antigen–antibody complexes, and paraproteins. These molecules may be cleared through two alternative procedures: centrifugal separation and membrane separation [37]. Extracorporeal therapies hemodialysis and therapeutic plasma exchange have been considered to remove cytokines in patients with sepsis and may be used in critically ill patients with COVID-19 [38]. The removal of cytokines may avoid damage to other tissues and organs. This can be achieved through four types of approaches: direct hemoperfusions using a neutro-macroporous sorbent, plasma adsorption using a resin after plasma is separated from whole blood, continuous kidney replacement therapy (CKRT) with hollow fiber filters of adsorptive properties, and high-dose CKRT with medium cut-off or high cut-off membranes [35].

## 4. Vitamin C and Its Potential for the Protection of Renal Injury in Patients with COVID-19

Vitamin C acts as an antioxidant to clear reactive oxygen and nitrogen species [39]. For example, it protects lung cells from oxidative damage [40]. Therefore, one important activity of vitamin C is to block oxidative stresses, therefore, reducing or preventing productions of reactive oxygen and nitrogen species derived from cellular activities in response to bacterial and viral infections. The viral infections may trigger cytokine storms and lead to increased oxidative stresses in cells and tissues [41].

Table 1 summarizes studies on using vitamin C to intervene AKI in animal models, which include rabbits and rats. These reports retrieved in the literature search demonstrate the uses of vitamin C to prevent and treat AKI before and after the establishment of renal injury model, respectively. Blood creatinine, urea, malondialdehyde, and reduced glutathione levels, the makers of renal functions and oxidative stresses, were measured in these studies [42,43,44,45]. The results showed that vitamin C protected the kidneys from injuries caused by external factors, and also facilitated repair after damage occurred. Vitamin C treatment shortens the repair time needed for the kidneys. At the same time, data of malondialdehyde and reduced glutathione have shown that vitamin C treatment can effectively increase antioxidant capacity in the blood, and thus protect against renal injury [45]. Vitamin C probably acts to remove reactive oxygen free radicals and prevent the accumulation of oxidation products, which cause damage to the kidneys. In some studies, pretreatment with vitamin C resulted in marked improvement in renal functions, manifested by significant decreases in plasma urea and creatinine levels and kidney tissue malondialdehyde levels [46].

**Figure 1:**
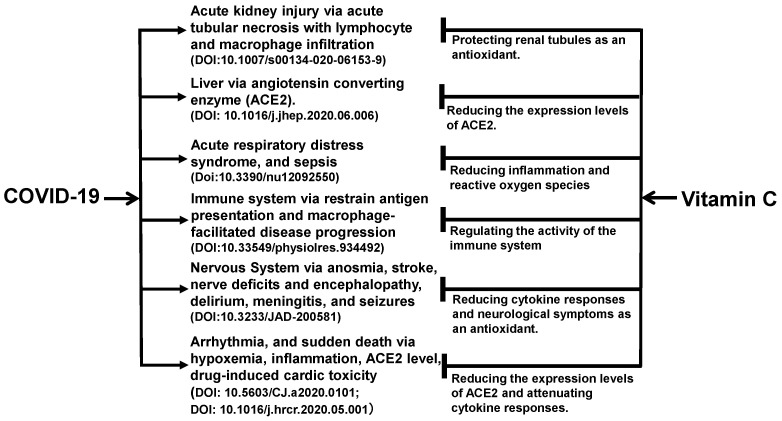
Ascorbic acid has been experimentally proven to ameliorate comorbid conditions in SARS-CoV-2-infected patients. Vitamin C appears to promote immune function and reduce inflammation and oxidative stress, and in turn, protect multiple organs in patients with COVID-19. As an antioxidant, vitamin C prevents acute necrosis mediated by immune cells in the kidneys. For the lungs, heart, liver, vitamin C reduces the expression levels of angiotensin converting enzyme 2 (ACE2) expression levels, which limits the binding of the viral particles to the cells. For the kidneys, it prevents the development of acute kidney injury. For the immune system, its autophagy-inducing mechanism impedes the severity of COVID-19 by producing interferons and decreasing the levels of inflammatory interleukins.

The benefits of vitamin C may not be limited to the kidneys. As shown in Figure 1, vitamin C acts as an antioxidant in the body to exert its effects on multiple organs and tissues of the body [47,48,49,50,51,52,53]. The angiotensin-converting enzyme 2 (ACE2) is a functional receptor for SARS-CoV-2 to enter host target cells [54]. ACE2 is widely found in human liver, lungs, kidneys, intestinal tract, and other organs [55]. For the lungs, heart, and liver, vitamin C reduces the expression levels of ACE2 expression levels, which limits the binding of the viral particles to the cells and protects these organs directly from the viral damage.

Vitamin C has a variety of pharmacological properties, antiviral, antioxidant, anti-inflammatory, and immunomodulatory effects, and is a potential treatment option for COVID-19 [56,57]. During the acute phase of infection, vitamin C levels in the plasma and white blood cells decrease due to increased metabolic demands. High-dose vitamin C supplementation helps to restore the plasma and white blood cell vitamin C levels. It appears to work by enhancing the function of immune cells and by its antioxidant properties [7]. Vitamin C can support a variety of cellular functions of the immune system, helping to maintain immunity. It supports epithelial barrier function against pathogens and promotes oxidative scavenging activity in the skin, thereby potentially protecting against oxidative stress from the environment [58,59,60]. Moreover, it can enhance the function and chemotaxis of phagocytes and neutrophils, phagocyte bacteria, produce reactive oxygen species, and eventually kill microorganisms [7]. In the early stage of COVID-19 infection, the early manifestations of cardiovascular disease are often accompanied by vascular endothelial dysfunction and organic lesions while oxidative stress and blood pressure can damage vascular endothelium [61]. The antioxidative stress characteristics of vitamin C may prevent and slow the onset of heart risk in patients at an early stage [62]. Elderly patients hospitalized with pneumonia or bronchitis who took vitamin C were at least 80% less likely to develop pneumonia, according to the data of a randomized trial [63]. Therefore, vitamin C protects multiple organs in patients with coronavirus infection. Table 2 summarizes the registered clinical trials that tested the use of vitamin C in the combat against COVID-19 at ClinicalTrials.gov on 20 June 2021.

In animal studies, vitamin C has been shown to protect and shorten the duration of treatment after kidney damage caused by harmful substances [45]. The virus directly acts on vascular endothelial cells, leading to endothelial inflammation, which affects several important organs and causes multiple organ failures. The antioxidant and anti-inflammatory effects of vitamin C may protect endothelial cells, and thus reduce the incidence of multiple organ failures [64]. In animal studies, the infection of H3N2 can kill mice that are deficient in vitamin C [41]. Here, vitamin C has been thought to be needed for anti-influenza virus responses in the early phase of infection, which involves the production of IFN-α/β. IFNs may promote virus clearance, lowing the number of virus-specific CD8+ and CD4+ T-cells [65].

The tolerable human upper intake level of vitamin C is 2000 mg/day per person in the general population [66]. The highest dose of ascorbic acid used in a rabbit study has been 250 mg/kg/day, which would be about 15,000 mg/day for a person weighing 60 kg [45]. Intravenous administration of vitamin C at 1.5 g/kg body weight is considered to be safe for clinical use [67]. One early clinical trial, NCT04264533 (shown in Table 2), planned to infuse 12 g of vitamin C twice a day for 7 days. We have not found a recommended dose of vitamin C to prevent kidney injury in SARS-CoV-2 infected patients. It is reasonable to think that these doses will depend on the routes of delivery (orally or intravenously) and probably age of the patients. The completion of those clinical trials shown in Table 2 probably will offer us more clues as what the routes of administration and doses of vitamin C will be to prevent kidney injury in patients with COVID-19.

**Figure 2:**
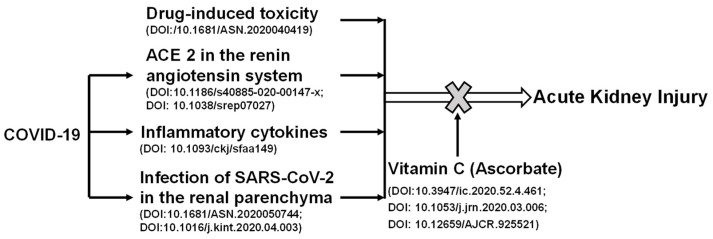
The positive role of vitamin C in acute kidney injury induced by chemical toxicity or COVID-19. Renal toxic chemicals and development of COVID-19 can cause acute kidney injury. SARS-CoV-2 viral particles enter cells via its interacting protein angiotensin converting enzyme 2 (ACE2). Viral infections lead to cytokine storm and cause hemodynamic changes through the high expression of ACE2. Vitamin C acts to reduce oxidative stresses and repair damage, and in turn, attenuates acute kidney injury. Vitamin C can reduce the expression of ACE2, which hinders the entry of virus into cells, and stabilizes blood pressure.

Recently, it has been shown that AKI has a high incidence in patients with severe COVID-19 [68]. Kidney involvement is associated with a poor prognosis. As shown in Figure 2, the potential mechanisms involved in renal injury during COVID-19 may include infection, direct invasion of renal parenchyma and hemodynamic instability secondary to renal injury, the inflammatory cytokine storm, and the use of nephrotoxic drugs. Renal toxic chemicals and development of COVID-19 can cause AKI. Vitamin C protects against the AKI induced by chemical toxicity or COVID-19. SARS-CoV-2 viral particles enter cells via its interacting protein ACE2. Viral infections lead to cytokine storm and cause hemodynamic changes through the high expression level of ACE2 [54]. Vitamin C acts to reduce oxidative stress and repair damage, in turn, attenuating AKI. Vitamin C can reduce the expression of ACE2, which hinders the entry of the virus into cells, and stabilizes blood pressure.

Coronavirus can cause dysfunction of ACE2, leading to the activation of the renin-angiotensin system, and ultimately to change blood pressure and aggravate excessive inflammatory responses [69]. One important autopsy report of a COVID-19 death showed acute protrusion of proximal tubule injury, but also peritubular erythrocyte aggregation and glomerular fibrin thrombosis with ischemic collapse [31]. This report also described endothelial injury, hemosiderin deposition, pigment-tube pattern, and inflammation associated with rhabdomyolysis. It is important to note that some of these patients had early evidence of AKI that could not be detected by routine tests (creatinine and/or urea nitrogen), which made it easy to overlook the possibility of subclinical renal injury [31]. Viral infections lead to CRS and cause hemodynamic changes through the high expression of ACE2. Vitamin C can reduce the expression of ACE2, which hinders the entry of the virus into cells, and stabilizes blood pressure. It reduces oxidative stresses, repairs damage, and in turn, attenuates AKI.

It is worthwhile noting that another common dietary antioxidant is vitamin E, which is found on cellular membranes as an antioxidant [70], and acts as a peroxyl radical scavenger to protect polyunsaturated fatty acids to go through autooxidation [71]. ClinicalTrails.gov includes one clinical trial, NCT04570254, to study antioxidant as adjuvant therapy to standard therapy in patients with COVID-19. Vitamin E has multiple isomeric forms and has activities beyond its function as an antioxidant [72]. Therefore, more studies are needed before we can pinpoint the role of vitamin E in the combat against kidney injury in patients with COVID-19.

## 5. Summary and Future Perspectives

As a micronutrient, vitamin C takes part in the body’s metabolism, and its antioxidant activity plays an important role in protecting the kidneys, lungs, and other organs. Patients with coronavirus infection in critical conditions have poor nutrition, indicating that adequate vitamin C uptake or supplementation should be considered for them, especially the elderly and patients with a variety of basic diseases. Adequate vitamin C provides protection to the kidneys and vital organs in the body against inflammation and oxidative stresses. At the same time, it contributes to the stability of the body’s metabolism and maintains a healthy state of the internal environment. Future studies could focus on the role of vitamin C in the regulation of renal cell functions, which have diverse structures and functions. In addition, vitamin C’s functions in the infections of other viruses should be studied in clinical settings.

## Figures and Tables

**Table 1:** Vitamin C’s protection function in the kidney injuries.

Animals	Reagents Used	Ascorbate Acid Dose	Test Time	Effective	Reference
Male albino rabbits (25, 5 groups)	Gentamicin, 80 mg/kg, im qd	250 mg/kg qd	26 days	Yes (*p* < 0.05)	[42]
Male Wistar rats (56, 7 groups)	Diazinon, 20 mg/kg Ceftriaxone, 100 mg/kg	100 mg/kg qd	28 days	Yes (*p* < 0.05)	[43]
Male Sprague-Dawley rats (25, 5 groups)	Colistin	200 mg/kg, bid	7 days	Yes (*p* < 0.05)	[44]
Male Sprague-Dawley rats (48, 6 groups)	Nevirapine, 200 mg/kg	250 mg/kg qd	28 days	Yes (*p* < 0.05)	[45]

**Table 2:** An updated list of registered clinical trials to test the use of vitamin C in the treatment of COVID-19 at ClinicalTrial.gov.

Start Date	Status	Identifier	Study Title
11-8-2018	Recruiting	NCT03680274	Lessening organ dysfunction with vitamin C
02-14-2020	Terminated	NCT04264533	Vitamin C infusion for the treatment of severe 2019-nCoV infected pneumonia
03-13-2020	Recruiting	NCT04323514	Use of ascorbic acid in patients with COVID 19
03-31-2020	Completed	NCT04328961	Hydroxychloroquine for COVID-19 post-exposure prophylaxis
04-16-2020	Completed	NCT04357782	Administration of intravenous vitamin C in novel coronavirus infection (COVID-19) and decreased oxygenation
04-16-2020	Active, not recruiting	NCT04354428	Treatment for COVID-19 in high-risk adult outpatients
04-20-2020	Withdrawn	NCT04347889	Preventing COVID-19 in healthcare workers with HCQ: A RCT
04-19-2020	Recruiting	NCT04370288	Clinical application of MCN (methylene blue, vitamin C, N-acetyl cysteine) for treatment of Covid-19 patients
05-2020	Not yet recruiting	NCT04363216	Pharmacologic ascorbic acid as an activator of lymphocyte signaling for COVID-19 treatment
05-25-2020	Recruiting	NCT04395768	International ALLIANCE study of therapies to prevent progression of COVID-19
05-30-2020	Suspended	NCT04334967	Hydroxychloroquine in patients with newly diagnosed COVID-19 compared to standard of Care
06-2020	Recruiting	NCT04401150	Lessening organ dysfunction with vitamin C—COVID-19
06-20-2020	Recruiting	NCT04468139	The study of quadruple therapy zinc, quercetin, bromelain and vitamin C on the clinical outcomes of patients infected with COVID-19
06-22-2020	Recruiting	NCT04335084	a study of hydroxychloroquine, vitamin C, vitamin D, and zinc for the prevention of COVID-19 infection
06-22-2020	Recruiting	NCT04334512	A study of quintuple therapy to treat COVID-19 infection
10-2020	Recruiting	NCT04344184	Early infusion of vitamin C for treatment of novel COVID-19 acute lung injury
11-18-2020	Recruiting	NCT04344184	SAFEty study of early infusion of vitamin C for treatment of novel coronavirus acute lung injury
